# Virtual treadmill technology (C-mill) in gait rehabilitation after stroke: A qualitative interview study

**DOI:** 10.1177/20556683261487182

**Published:** 2026-09-07

**Authors:** Kristiansen Sara, Lahelle Falck Andreas, Wouda Matthijs Ferdinand

**Affiliations:** 1 Department of Rehabilitation Science and Health Technology, Oslo Metropolitan University, Oslo, Norway; 2 Active Clinic, Lillehammer, Norway; 3 Centre for Research and Education, Sunnaas Rehabilitation Hospital, Nesoddtangen, Norway

**Keywords:** stroke, rehabilitation, C-mill, motivation, patient experience

## Abstract

**Background:**

In stroke rehabilitation, VR-based technologies like the C-Mill treadmill combine physical and cognitive training to enhance motivation and motor learning. While quantitative studies show functional benefits, little is known about patients’ experiences.

**Aim:**

This study aims to explore stroke survivors’ subjective experiences of motivation during C-Mill training.

**Method:**

A qualitative design was employed. Seven inpatients at Sunnaas Hospital who had engaged in gait rehabilitation with C-Mill participated in semi-structured interviews. Data were transcribed verbatim and analyzed using systematic text condensation.

**Results:**

One overarching theme - motivation - was identified, comprising two subthemes: intrinsic and extrinsic motivation. The patients described how the C-Mill fostered intrinsic motivation by providing challenging, meaningful, and engaging activities that reflected everyday-life situations. They experienced that the system encouraged them to push their limits and attempt tasks they might otherwise hesitate to try, while still feeling supported and safe. Additionally, the technology enhanced extrinsic motivation through engaging feedback mechanisms, including gamification elements, and the visualization of performance statistics.

**Conclusion:**

C-Mill appears to constitute a promising adjunct to conventional rehabilitation by strengthening motivation. Its technological features, including safety scaffolding, gamification, and task variability, appeared to facilitate engagement and promote motivation.

## Introduction

Stroke is a leading cause of long-term disability, often resulting in persistent impairments, including reduced mobility, aphasia, depression, and cognitive decline, that substantially limit functional independence and quality of life.^1^ Effective rehabilitation is therefore critical, yet sustaining engagement in often repetitive and demanding therapy remains a major challenge. Motivation is a key determinant of rehabilitation success, but it is frequently difficult to maintain over time. Innovative approaches, such as C-Mill, a treadmill training combined with virtual reality technologies, can address functional deficits and enhance engagement. However, their impact on patients’ motivation is not yet well understood. The present study aims to cover this knowledge gap.

Among stroke survivors in Norway, about one-third regain full or near-full function within the first year post-stroke, while one-third live with long-term disability.^2^ Several RCT’s have shown that early and intensive task-oriented rehabilitation enhances functional regain after stroke.^3^ Moreover, motivation has been shown to serve as a crucial link between cognitive processes and motor performance, making it a key factor in determining rehabilitation outcomes.^4^ According to the American Congress of Rehabilitation Medicine (ACRM) rehabilitation is a dynamic process including interventions to optimize functioning and reduce disability in individuals with health conditions interacting with their environment.^5^ Acute rehabilitation begins as early as medically feasible following stroke onset, while adaptation to new life circumstances is a longer-term process that continues well beyond the acute phase.

### Theoretical framework

To investigate how patients’ motivation can be affected during rehabilitation using C-Mill, we considered neuroplasticity, self-determination theory (SDT) and motivation theory as relevant theoretical perspectives for interpreting and illuminating the findings. These perspectives emerged as relevant through the analytical process following data collection and are presented in the following sections.

Neuroplasticity, the brain’s ability to reorganize, is central to recovery. Contrary to earlier assumptions, adult brains can form new synapses, reorganize activity, and even generate neurons.^6^ After stroke, this plasticity allows individuals to relearn lost functions through targeted activity and training, with early and intensive rehabilitation optimizing these processes. Kleim and Jones outline principles of experience-dependent plasticity that are particularly relevant for technological solutions like the C-Mill.^6^ Active practice strengthens neural networks (“Use It and Improve It”), while task-oriented exercises ensure that training is relevant to functional goals (“Specificity”). Moreover, repeated and intensive practice reinforces neural pathways and supports motor learning, especially when exercises are meaningful and engaging (“Repetition Matters,” “Intensity Matters,” and “Salience Matters”), which can enhance motivation and adherence. Importantly, skills acquired during training can generalize to everyday activities, allowing, for instance, gait adjustments practiced on the C-Mill to transfer to daily walking tasks (“Transference”). By integrating these principles, C-Mill training can effectively support engagement, motivation, and recovery in stroke survivors.

Ryan and Deci distinguish between two main forms of motivation: intrinsic and extrinsic, both of which influence engagement in activities, including rehabilitation.^7^ Intrinsic motivation refers to performing an activity because it is inherently interesting, challenging, or satisfying. It is the most autonomous form of motivation and is closely linked to the fulfillment of the three basic psychological needs in Self-Determination Theory (SDT): autonomy, competence, and relatedness. When these needs are met, individuals are more likely to experience increased intrinsic motivation, leading to sustained effort and better rehabilitation outcomes. VR technologies such as C-Mill can provide patients with engaging and challenging experiences that enhance feelings of autonomy and competence, thereby strengthening intrinsic motivation.

Extrinsic motivation is driven by external factors, such as rewards, recognition, or specific goals. Ryan and Deci describe a spectrum of extrinsic motivation ranging from external regulation to more internalized forms.^7^ External regulation involves performing an activity to gain rewards or avoid punishment, whereas introjection involves acting to avoid internal conflicts, such as guilt or shame. More internalized forms occur when individuals identify with the value of an activity, making extrinsic motivation more autonomous and effective for long-term engagement. In rehabilitation, extrinsic motivation may drive patients to improve physical function, regain independence, or achieve specific goals.

SDT is therefore particularly relevant for understanding motivation in rehabilitation. By supporting both intrinsic and extrinsic motivational factors, VR technologies like C-Mill can enhance patients’ autonomy, engagement, and sustained effort, contributing to more successful rehabilitation outcomes.

### Previous research

According to national^2^ and international guidelines,^8^ treadmill training with body-weight support is recommended for patients with poor or absent walking ability. Combined treadmill and conventional gait training may benefit those with reduced but preserved function. These guidelines also recommend early, intensive, task-oriented training, involving physiotherapy, occupational therapy, and everyday activities.^2^

Over the past decades, a growing use of technology in stroke rehabilitation has improved access to therapy, enhanced patient engagement, and promoted better functional outcomes. New rehabilitation technologies have been introduced to the market, including the C-Mill treadmill (Motek Medical, Houten, the Netherlands). C-Mill is an advanced training and rehabilitation system developed to improve balance, gait, and gait adaptability in individuals with walking and balance problems.

Most research on C-Mill is quantitative, particularly randomized controlled trials (RCTs), focusing on safety, feasibility and clinical outcomes.^9,10^ Heeren et al. reported that post-stroke survivors experienced increased confidence, walking speed, and endurance after C-Mill training, with some noting generalization to other daily activities such as cycling and stair climbing.^11^ Pullia et al. found that C-Mill training improved gait and balance in patients with Parkinson’s disease, showing greater gains than conventional physiotherapy, suggesting potential benefits across neurological conditions.^10^ Wang et al., however, did not find significant differences between intervention and control groups, reflecting variability in outcomes and the need for further investigation.^9^ Only one qualitative study has explored stroke survivors’ experiences with this type of gait training, showing positive feedback from all participants.^11^

Maintaining motivation throughout stroke rehabilitation is essential for achieving long-term recovery goals, as it may foster persistence, engagement, and a positive outlook on progress.^4^ Studies on virtual reality (VR)-based rehabilitation highlight how technology can enhance motivation, engagement, and variation in training. For example, Pallesen et al. found that VR for arm training after stroke increased patient motivation and engagement, while therapists emphasized the importance of individual adaptation and support.^12^ Similarly, Moan et al. reported that treadmill-based VR training was perceived as motivating and beneficial, especially when tasks were adapted to patients’ individual abilities.^13^ Chen et al. also found that VR rehabilitation increased engagement and perceived functional improvements, though technical challenges and the need for individualized adjustment were highlighted.^14^ These findings are relevant for C-Mill, which integrates physical activity with virtual elements to engage patients during gait and balance training.

Overall, previous research suggests that technology-assisted rehabilitation, including treadmill and VR-based systems, can enhance motivation, engagement, and functional improvements. However, the qualitative evidence on stroke survivors’ subjective experiences with C-Mill is sparse.^11^ There is therefore a need to better understand how patients experience this technology in clinical practice.

### Aim and research question

This study addresses this gap by exploring stroke survivors’ perspectives on using C-Mill for gait and balance rehabilitation. Specifically, it aims to generate new knowledge about how VR-based treadmill training is perceived in a clinical rehabilitation context and how it may influence motivation and engagement during gait training. Accordingly, the study addresses the following research question: *How do stroke survivors experience the use of C-Mill technology in relation to motivation and engagement during gait training*?

## Methods

### Design

The aim of this study was to explore stroke survivors’ experiences and perceptions of using the C-Mill in post-stroke rehabilitation. A qualitative research design using semi-structured interviews was applied. The study is situated within an interpretive paradigm, in which knowledge is understood as co-constructed through individuals’ interpretations of their experiences.^15^ This paradigm draws on philosophical traditions such as phenomenology, emphasizing lived experience, and hermeneutics, highlighting interpretation and context. However, the study is not positioned as strictly phenomenological or hermeneutical, but adopts a pragmatic qualitative approach, allowing flexibility in the selection of theoretical perspectives and analytical methods appropriate to the research aim. This allowed for an iterative analytic process using systematic text condensation as the method of analysis, as well as a flexible and emergent use of theory in interpreting the findings, which ultimately came to be primarily informed by SDT and the established understanding of the brain as plastic.

The study followed Brinkmann and Kvale’s seven-step framework for interview research to ensure methodological rigor^16^ and was performed in line with standard qualitative reporting conventions such as those recommended by the Consolidated Criteria for Reporting Qualitative Research (COREQ).^17^

#### Context of study

**Figure 1.**
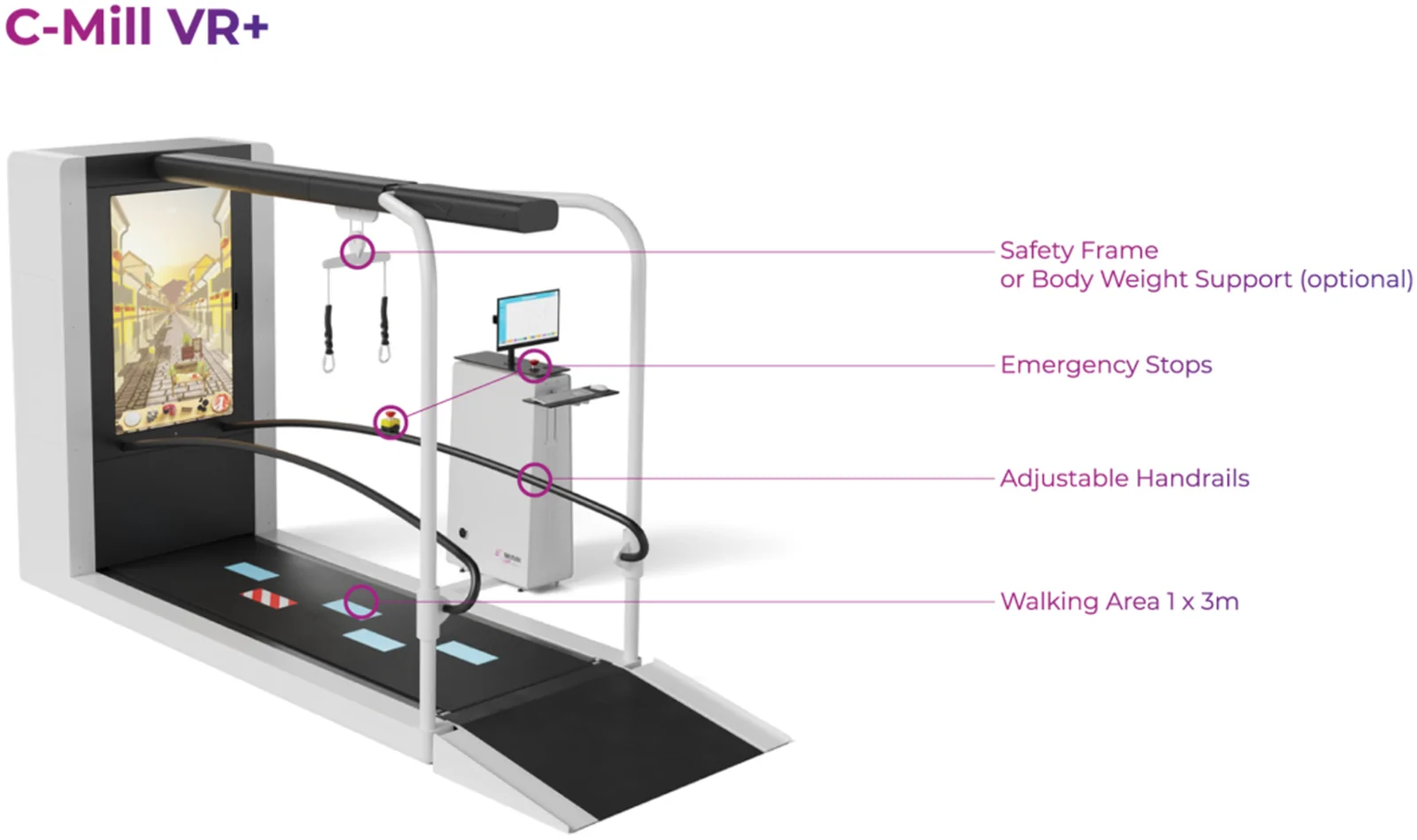
C-mill (motek medical).

The data collection of this study was conducted while the patients were undergoing rehabilitation at Sunnaas Hospital, where they used the C-Mill technology as a part of their overall rehabilitation program. The C-Mill system combines a treadmill with an integrated force plate and a projection system that displays visual targets and obstacles directly on the belt (Figure 1). This allows patients to practice realistic and varied walking challenges in safe surroundings.

The purpose of C-Mill is to provide an environment for dual-task training, requiring patients to perform a range of gait patterns, such as varying step lengths and navigating obstacles. By simulating these complex walking scenarios, the system offers engaging and functionally relevant challenges that can help sustain patients’ attention and participation during walking training. The system provides direct and measurable feedback on performance and can therefore be used both for assessment and targeted training of balance and gait. C-Mill also enables balance assessments testing both static and dynamic balance (Motek Medical B.V., 2025).

The C-Mill interactive treadmill enables safe assessment of walking adaptability.^18^ In stroke survivors, gait training using a C-Mill appears to induce a doubling in the number of steps compared to traditional walking exercise.^19^

### Participants

The participants were stroke survivors who underwent rehabilitation at Sunnaas Hospital. They had varied training programs during their stay, consisting of different forms of exercise, such as C-Mill training. Inclusion criteria were stroke survivors who were able to stand (with or without assistive devices) and who had completed at least two C-Mill training sessions. Exclusion criteria included severe aphasia, non-Norwegian speakers, or other serious comorbidities. The sample included four women and three men, aged 49–76 (Table 1). Eligible patients were identified by the stroke unit physiotherapist, informed about the study by a project team member, and invited to provide written consent. Interviews were scheduled after consent, and the patients’ physicians were notified of their participation. In this study, participants were recruited until sufficient depth and variation of information had been achieved across the interviews.

**Table 1.** Participants characteristics.

Participant	Gender	Age (years)	Stroke (type)	Time since injury (years)	Training sessions (n)
1	Woman	52	Hemorrhagic	4	3
2	Man	76	Hemorrhagic	2	2
3	Woman	Unknown^a^	Unknown^a^	2	6
4	Man	52	Ischemic	7	4
5	Man	66	Ischemic	4	2
6	Woman	49	Ischemic	2	5
7	Woman	59	Ischemic	1	7

^a^Not recorded in available data.

### Data collection

In line with the research question and study design, semi-structured, theme-based in-depth interviews^20^ were conducted using an encrypted online audio recording service (Nettskjema (https://nettskjema.no/)). A peer consultant at Sunnaas Hospital contributed to develop an interview guide to incorporate the user perspective. A pilot interview was conducted to test flow and wording, resulting in minor adjustments.

The first author conducted the interviews at Sunnaas Hospital in the late afternoon/evening to accommodate patients’ schedules and training plans, averaging 30–40 minutes, allowing sufficient time to explore all relevant aspects of patient experiences. All recordings were securely stored in a secure platform for storing and analyzing sensitive research data (TSD (About TSD - University of Oslo)), offering encrypted storage, controlled access, and compliance with data protection standards. After each interview, the researcher recorded reflective notes in a digital logbook to support analysis and interpretation.

### Data analysis

To prepare the interview material for analysis, audio recordings were transcribed. Transcription transforms oral conversation into written text, which serves as the foundation for analysis, while requiring attention to technical, interpretative, and ethical considerations.^16^ A multilingual speech recognition model was used for initial transcription (Autotekst (https://www.uio.no/english/services/it/video-sound/autotekst/)). NVivo (Nvivo software (https://www.uio.no/english/services/it/research/data-collection-and-analysis/nvivo/index.html)) was used to structure and manage the analysis, allowing organization, coding, and cross-referencing of qualitative data in line with Malterud’s method.^20^

Data analysis was led by the first author, who was responsible for organizing the analysis process, and managing the organization of the coded material. However, the analysis was conducted through an iterative and collaborative process involving all authors. The second and third authors participated in regular meetings throughout the analysis, where codes, meaning units, and interpretations were continuously discussed and refined. These discussions included reflections on theoretical perspectives and contextualized our findings within a rehabilitation perspective. More specifically, the transcribed data were analyzed using Malterud’s four-step method for systematic text condensation, a pragmatic approach for thematic analysis of qualitative data. Initially, the empirical material was analysed with an inductive orientation, allowing patterns and themes to emerge from the data.^20^ This was followed by an abductive process^21^ in which emerging insights were iteratively interpreted in dialogue with existing theories addressing motivation. The steps in systematic text condensation include: (1) gaining an overall impression and identifying preliminary themes, (2) identifying meaning units and coding relevant data, (3) condensing coded material into subgroups and summarizing key findings, and (4) synthesizing the condensed data into coherent descriptions, concepts, and results.^20^ Recurrent themes across interviews were coded into the category motivation. Subcategories were then created intrinsic and extrinsic motivation, and condensed into illustrative statements for each participant. These condensates were later synthesized into analytical texts supported by illustrative quotations, forming the basis for the results and discussion.

## Research team and reflexivity

The first author is an occupational therapist with a master’s degree in rehabilitation and habilitation. The second and last authors are physical therapists with clinical and research experience in neurological rehabilitation. The second and last authors have previously applied SDT as a theoretical framework in their research. To enhance the trustworthiness of the study and address the potential risk of theoretical confirmation bias, the analysis followed an abductive approach.^21^ Thus, SDT informed the interpretation of the findings while the analysis remained attentive to perspectives and themes that extended beyond the theoretical framework.

### Ethical considerations

The study was conducted in accordance with the principles of the Declaration of Helsinki and was approved by The Norwegian Agency for Shared Services in Education and Research (#388134). Ethical approval was also obtained from the Data Protection Officer at Sunnaas Hospital.

## Results

The analysis contributed to the development of knowledge regarding the participants’ subjective experiences related to the use of the C-Mill in gait rehabilitation after stroke. The findings are presented as analytical text based on the interviews and are illustrated with direct quotes from the participants. A thematic analysis of the main theme, subthemes, and categories is shown in Table 2.

**Table 2.** Thematic analysis.

Main theme	Sub-theme	Categories
Motivation	Intrinsic motivation	Variation and C-Mill as a supplement
		Transferability
		Perceived change or improvement
	Extrinsic motivation	Gamification, feedback and statistics

### Motivation

A recurring experience among the participants was whether they perceived the C-Mill as motivating. A distinction has been made between intrinsic and extrinsic motivation in order to clarify the different factors that may influence motivation.

### Intrinsic motivation

A key aspect of intrinsic motivation in this context is the element of variation, both in the selection of games offered by the C-Mill and in the different ways these games can be played, as well as in the diversity of training modalities within gait rehabilitation. Intrinsic motivation also relates to the extent to which participants perceived the C-Mill as promoting transferability and relevance to everyday challenges, and whether they felt it contributed to improvement or recovery. Repetitive exercises can quickly become monotonous, and participants emphasized that the enjoyable and playful nature of the C-Mill made training more engaging. When combined with other rehabilitation methods, it was perceived as an effective and valuable supplement. Overall, participants experienced the C-Mill as more motivating than other forms of gait training, particularly because it was fun, functional, and reflected real-life obstacles they encountered in daily activities.

#### Variation and C-Mill as a supplement

Several participants emphasized that the C-Mill provided a varied exercise experience, which served as a key source of motivation. The wide range of games, adjustable difficulty levels, and visual stimuli made the training both challenging and engaging. The combination of physical movement and cognitive demands was described as enjoyable and unique: the dynamic treadmill, together with visual tasks, required focus and coordination, which enhanced both motivation and training effect.

An important prerequisite for engaging in these challenging tasks was the sense of safety provided by the equipment. The harness, emergency stop, and handrails created a secure environment that allowed participants to push their limits without fear of falling. As participant (P) 2 explained: “*I feel safe because I’m hanging from a strap in the ceiling, so there’s no chance of falling. I can really go for it and do anything without fear.*” This security enabled participants to fully engage in demanding exercises such as stepping over obstacles and performing zigzag patterns.

Participants reported that C-Mill training increased attention, created a sense of mastery, and contributed positively to self-confidence and perceived progress. Variation was also linked to the ability to adapt sessions to individual ability and daily form, allowing participants to take an active role in their rehabilitation. Many described the C-Mill as more motivating than traditional gait training, which was often perceived as repetitive or monotonous. The playful, game-based nature of the training was highlighted as a refreshing alternative that made exercise feel less like a chore and more like an enjoyable challenge. P1 expressed: “*I really believe in things that increase motivation. Variety increases my motivation, and it’s a fun supplement*.” P5 added: “*I find the C-Mill motivating, and I look forward to every training session. One of the reasons is that I never really know what to expect, because there are so many different tasks*.”

Furthermore, participants viewed the C-Mill as an effective supplement to other forms of rehabilitation. Its combination of functionality, safety, and enjoyment made it a valuable addition rather than a replacement for conventional training. As P1 stated: “*It’s not something you have to do, it’s something you want to do.”*

#### Transferability

Several participants experienced the training effects of the C-Mill as directly transferable to challenges encountered in everyday life. They described the training as functional and meaningful, not merely as relearning how to walk, but as practicing movements that resembled real-life obstacles. As P7 explained: *“C-Mill reflects my everyday challenges, stepping on or over things, avoiding objects, and moving back and forth. It feels very relevant.”*

This sense of transferability contributed to a stronger perception of purpose and mastery, as participants perceived that the training prepared them for daily mobility tasks. Some also recognized its limitations, noting that C-Mill does not fully replicate variations in surface texture or elevation found in real-life environments. Still, the participants emphasized that it provided a high degree of relevance and confidence in dealing with practical movement challenges.

Overall, the findings suggest that C-Mill training enhances motivation by offering functional, realistic, and safe practice that participants perceived as directly applicable to their everyday lives.

#### Perceived change or improvement

All participants reported some form of improvement during their rehabilitation stay, and several attributed this progress to training with the C-Mill. They described perceived gains in walking speed, confidence, and functional ability, noting that the treadmill encouraged continuous movement and quick reactions. As P6 explained: *“Usually I would stop in front of an obstacle, but on the C-Mill you don’t have time, you just have to step over. That’s one of the effects I’ve noticed the most: that I dare more.”*

Participants also emphasized psychological benefits, such as increased balance confidence and reduced fear of falling. P1 reflected: *“C-Mill made me realize that my balance is better than I thought. I’ve had a lot of fear of falling, but now I feel more motivated to practice.”*

While some highlighted that their improvement was the result of the overall rehabilitation program, many viewed C-Mill as a significant contributing factor that enhanced motivation, self-efficacy, and a sense of mastery.

### Extrinsic motivation

Extrinsic motivation relates to participants’ experiences of the C-Mill’s gamification elements, and visual feedback systems.

Participants also described being motivated by the game-like elements of C-Mill, including points, scores, and visual progress graphs. Several expressed that these features made the training engaging and enjoyable, often to the extent that they “forgot they were exercising” because it felt like play rather than rehabilitation.

#### Gamification, feedback and statistics

Participants consistently highlighted the motivating effect of gamification elements in C-Mill, such as points, scores, and game-based tasks. These elements encouraged them to perform movements they might otherwise avoid, and often made training feel like play rather than exercise. P1 explained: *“I’m triggered by the points, not to compete with others, but to beat the game itself. It makes me want to achieve it and move my leg in ways I normally wouldn’t.”*

Several participants described how games, such as moving a basket to catch virtual fruit, prompted them to take side steps and more challenging movements, enhancing engagement and functional practice. They also reported that gamification increased self-competition and confidence, motivating them to improve on previous attempts. P7 noted: *“You get eager to score points. It boosts confidence and makes you want to do better than last time. You end up challenging yourself more than you thought you could.”*

The participants also valued the real-time statistics and visual feedback provided by C-Mill, including graphs showing weight distribution or leg usage. These features allowed them to track progress, verify improvement, and increase self-awareness. P3 reflected: *“Seeing the graphs showing how I use both legs equally is inspiring. I can see what I do right or wrong, and it helps me learn and improve.”*

P7 reported that the safety harness could occasionally interfere with balance, and described it as restrictive during movement: *“The harness pushes you a little, so you lose some balance. It interferes a bit.”* Participants 4 and 7 also noted that fitting the harness was time-consuming, which was considered impractical, particularly when several participants were scheduled to use the C-Mill consecutively. P5 reported that the handrails could occasionally obstruct movement, especially when the affected arm came into contact with them, resulting in loss of balance.

Overall, gamification and visual feedback contributed to a more engaging, motivating, and instructive training experience, allowing participants to enjoy the exercises, compete with themselves, and see tangible evidence of improvement despite some practical drawbacks such as the harness occasionally restricting movement and the time required to fit it.

## Discussion

The participants in this study perceived C-Mill as an engaging and motivating form of gait training. Several participants reported increased self-efficacy and enjoyment from experiencing progress, which contributed to their intrinsic motivation. Other factors including feedback from healthcare professionals, technological features of C-Mill, and gamification also played a significant role in maintaining extrinsic motivation throughout the training. Safety emerged as a central theme, with harnesses, emergency stops, and handrails contributing to a sense of security. Several participants noted that C-Mill training had enhanced their confidence and motivation to continue treadmill training beyond the rehabilitation setting.

### Intrinsic motivation

According to the participants, C-Mill enhances intrinsic motivation during gait training, which aligns with SDT.^7^ SDT posits that intrinsic motivation emerges when the psychological needs for autonomy, competence, and relatedness are satisfied. Several participants described feeling a sense of control and ownership over their training, as they could adjust difficulty levels and select tasks, supporting autonomy. Competence was reinforced through repeated success in challenging exercises, gamified tasks, and immediate feedback, while relatedness was supported through interactions with therapists and group training. This suggests that C-Mill supports psychological needs in ways that can enhance engagement and adherence to rehabilitation.

The role of variation within a training session was emphasized by the participants as a key factor for intrinsic motivation. Participants highlighted that the combination of physical and cognitive tasks, interactive games, and visual stimuli made exercises more engaging and unpredictable. Perceived safety can be understood as a prerequisite for motivation, as reducing fear and anxiety enabled participants to engage more fully with challenging tasks and focus on skill acquisition. Safety features such as the harness, handrails, and emergency stop were repeatedly emphasized as critical for participants’ willingness to challenge themselves. These features not only prevented injury but also created a psychologically safe environment, enabling participants to attempt movements they might otherwise have avoided on a conventional treadmill. While Ryan and Deci do not explicitly discuss safety as a form of extrinsic motivation, in this context, safety functions can be seen as supportive scaffolds that reduce anxiety and promote engagement. By mitigating fear of falling, participants could focus on skill acquisition, enhancing both motivation and motor learning. Moreover, stress and fear can interfere with the brain’s ability to adapt and learn, affecting the hippocampus and prefrontal cortex while promoting more rigid, survival-focused responses.^22–24^ As motor and cognitive relearning relies on neuroplasticity, feeling psychologically safe may therefore be a crucial prerequisite for both motivation and functional recovery.

According to Kleim and Jones, principles such as salience, specificity, and repetition are crucial for experience-dependent neuroplasticity.^6^ The salience principle, which emphasizes the importance of meaningful and relevant tasks, aligns with participants’ reports that C-Mill activities resembled everyday challenges, thus increasing motivation. Variation and novelty appear to support both engagement and learning, consistent with findings by Pallesen et al. and Moan et al., who reported that VR-based rehabilitation with varied, game-like tasks enhanced motivation and task performance.^12,13^

### Transferability and functional relevance

Participants experienced C-Mill as functionally relevant and transferable to everyday life. Many described obstacle navigation on C-Mill as comparable to challenges at home or outdoors, such as stepping over objects left on the floor. Some participants reported that it could be difficult at times to find the appropriate step length, and the treadmill surface occasionally felt distracting during walking. Although C-Mill obstacles are flat and do not replicate elevation changes, the overall experience was perceived as meaningful and directly applicable. This resonates with the transference principle described by Kleim and Jones, which suggests that skills acquired in one context can generalize to other, related contexts.^6^ The participants’ increased confidence, willingness to attempt previously avoided tasks, and perceived improvements in balance illustrate how C-Mill may support functional gains beyond the training environment.

The perceived functional relevance of C-Mill aligns with principles of task-oriented training and motor learning.^25,26^ Task-oriented approaches emphasize repetitive, goal-directed movements that are meaningful and relevant, which are thought to promote neuroplastic changes and skill acquisition. Participants’ experiences suggest that C-Mill may provide the type of goal-directed, high-repetition practice that is central to motor learning, supporting both skill development and confidence in daily mobility.

### Extrinsic motivation: Gamification and feedback

Gamification and feedback were also important motivators. Interactive games and point systems encouraged participants to engage in movements they might otherwise avoid, creating a sense of play and challenge. Many described “forgetting” that they were exercising due to the gamified environment, suggesting that gamification can effectively transform demanding rehabilitation tasks into enjoyable activities. This aligns with literature indicating that game-like elements increase adherence and motivation in rehabilitation.^12^

Visual feedback, including graphs and statistical data, supported both competence and self-efficacy, providing participants with concrete evidence of progress. SDT suggests that perceived competence strengthens intrinsic motivation, and participants’ experiences confirm that feedback helps them recognize improvement and adjust performance. Feedback also supports motor learning, consistent with Shumway-Cook et al., who highlight the importance of both intrinsic and extrinsic feedback for skill acquisition and correction of errors.

Although this study focused on the C-Mill technology, the mechanisms that supported motivation, confidence, and functional relevance might be related to general principles of effective rehabilitation, such as task variability, gamified engagement, immediate feedback, and a safe training environment. Rather than features unique to one system. These key elements are present in several digital rehabilitation technologies, including VR-based balance systems (variability, gamification), robotic treadmills (protected training environment), and sensor-driven gait trainers (immediate feedback). Therefore, the findings in this study have transferability to other technologies that share these characteristics, suggesting that the observed motivational and functional benefits could extend beyond the context of C-Mill. The underlying principles supporting motivation and functional engagement may have transferability to other patient populations and diagnoses as well, indicating that the benefits observed with C-Mill and comparable technologies could be applicable beyond the specific population studied.

This study explored participants’ experiences with C-Mill training, highlighting motivation, perceived competence, safety, and transferability to daily life as central themes. Overall, participants found C-Mill engaging, motivating, and functionally relevant, indicating its potential to support both motor learning and neuroplasticity in rehabilitation contexts.

## Methodological considerations

It is important to acknowledge the potential for social desirability bias, as participants were interviewed during their inpatient stay by a researcher affiliated with the clinical research team, which may have encouraged more favorable responses.

The trustworthiness of this study was evaluated using the concepts of validity, objectivity, and transferability as described by Kvale and Brinkmann.^16^ However, member checks and audit trials were not conducted. The study included seven participants. In qualitative research, sample size is guided by information power rather than statistical representativeness. According to Malterud et al., a smaller sample is sufficient when the study has a narrow aim, a relatively homogeneous sample, and rich, relevant data.^27^ As all participants had experience with the same intervention and provided detailed accounts of their experiences, the sample was considered adequate to address the study aim. A potential limitation is that all participants were recruited from Sunnaas Hospital, and those who agreed to participate may have had more positive attitudes towards research and the C-Mill intervention than those who declined. This may have introduced selection bias. However, two of the three researchers had no affiliation with Sunnaas Hospital, reducing the risk of allegiance bias and strengthening the independence of the analysis.

Complete objectivity is neither possible nor expected in qualitative research, as researchers’ pre-understanding inevitably influences the research process.^16^ The first author’s interest in rehabilitation technology may have influenced data collection and interpretation. To enhance reflexivity, this potential influence was continuously considered throughout the study, and both positive and negative experiences with the C-Mill were included in the analysis.

The aim of this study was not statistical generalisation but to provide an in-depth understanding of participants’ experiences. Although the findings are based on a small sample, they may have transferability to similar rehabilitation settings, particularly given the limited qualitative research exploring patients’ experiences with the C-Mill.

### Clinical implications and future research

Participants frequently compared their experiences with C-Mill to conventional gait training, describing greater variation, engagement, and motivation than with standard treadmills or walking exercises. The Norwegian stroke rehabilitation guidelines similarly emphasize the importance of varied, task-oriented, and high-repetition gait training to support improvements in balance, mobility, and endurance.^2^ The C-Mill’s combination of gamified, interactive tasks and repetitive gait practice may complement conventional therapy by supporting adherence and functional outcomes.

This study shows that the C-Mill can be a valuable tool for clinicians in gait rehabilitation of stroke survivors. By providing task-specific, repetitive, and functionally relevant exercises in a safe and engaging environment, C-Mill addresses both intrinsic and extrinsic motivators. Participants’ experiences suggest that this combination may enhance both skill acquisition and confidence, promoting transfer to everyday activities. Future research could explore the longitudinal effects of C-Mill training, including objective measures of gait, balance, motivation and adherence, as well as the relative contribution of gamification, feedback, and safety features to overall outcomes. In addition, C-Mill is a costly technology that is currently limited to a small number of rehabilitation centres with specialized personnel. Further research examining cost–benefit, accessibility, and feasibility would therefore be valuable to strengthen the evidence base for the use of this type of technology in rehabilitation. However, as several participants highlighted limitations related to the distracting treadmill surface and the flat representation of obstacles compared to real-world environments, further refinement might be needed to enhance ecological validity and transfer to everyday walking situations.

## Conclusion

This study provides valuable insights into stroke survivors experiences with C-Mill in gait training and its potential to inform clinical practice. Participants consistently described C-Mill as engaging, motivating, and more enjoyable than conventional gait training, highlighting the importance of both intrinsic and extrinsic motivational factors. Gamification elements, point systems, and visual feedback enhanced mastery and focus, allowing patients to train longer and with greater engagement, while safety features created a secure environment in which patients felt confident to attempt challenging movements.

While C-Mill shows promise as a supplemental or integrated tool in clinical settings, further research is needed to evaluate long-term effects, compare feedback modalities, and establish protocols for routine implementation. Nevertheless, C-Mill appears to be a promising adjunct to conventional rehabilitation by enhancing motivation, as its technological features - such as safety scaffolding, gamification, and task variability - facilitate engagement and promote participant motivation.
